# Supplementary material for: Empirical methods for controlling false positives and estimating confidence in ChIP-Seq peaks
Source: BMC Bioinformatics. 2008 Dec 5;9:523. doi: 10.1186/1471-2105-9-523 (PMC2628906; doi:10.1186/1471-2105-9-523)
Supplement: Additional File 1 — A variety of html documents from the USeq web site detailing the available applications, their best usage, output file type descriptions, command line menus, etc. [file 1471-2105-9-523-S1.zip › USeqUserGuides/JavaDoc.zip!/JavaDoc/util/gen/CubicSpline.html]

CubicSpline


|  |  |  |
| --- | --- | --- |
| **Overview** \| **Package** \| **Class** \| **Use** \| **Tree** \| **Deprecated** \| **Index** \| **Help** | |  |
| **PREV CLASS**   **NEXT CLASS** | **FRAMES**    **NO FRAMES**     **All Classes** |  |
| SUMMARY: NESTED \| FIELD \| CONSTR \| METHOD | DETAIL: FIELD \| CONSTR \| METHOD |  |


---


## util.gen Class CubicSpline

```
java.lang.Object
  util.gen.CubicSpline
```

---

``` public class CubicSpline extends java.lang.Object ```

For calculating cubic splines of incremental data, interpolation, from http://www.frank-buss.de/CubicSpline.java

---

**Constructor Summary**

`CubicSpline(double[] y)`
  
          The constructor calculates the second derivatives of the interpolating function
at the tabulated points xi, with xi = (i, y[i]).


| **Method Summary** | |
| --- | --- |
| `double` | `interpolate(int startIndex, double dist2NextPoint)`             Returns a cubic-spline interpolated value y for the point between point (n, y[n]) and (n+1, y[n+1), with t ranging from 0 for (n, y[n]) to 1 for (n+1, y[n+1]). |
| `static void` | `main(java.lang.String[] args)` |

**Methods inherited from class java.lang.Object**

`equals, getClass, hashCode, notify, notifyAll, toString, wait, wait, wait`

**Constructor Detail**

### CubicSpline

```
public CubicSpline(double[] y)
```

:   The constructor calculates the second derivatives of the interpolating function
    at the tabulated points xi, with xi = (i, y[i]).
    Based on numerical recipes in C, http://www.library.cornell.edu/nr/bookcpdf/c3-3.pdf .

    **Parameters:**: `y` - Array of y coordinates for cubic-spline interpolation.


**Method Detail**

### interpolate

```
public double interpolate(int startIndex,
                          double dist2NextPoint)
```

:   Returns a cubic-spline interpolated value y for the point between
    point (n, y[n]) and (n+1, y[n+1), with t ranging from 0 for (n, y[n])
    to 1 for (n+1, y[n+1]).

    :   **Parameters:**: `startIndex` - The start index/ point.: `dist2NextPoint` - The distance to the next point (0..1). **Returns:**: A cubic-spline interpolated y value.

---


### main

```
public static void main(java.lang.String[] args)
```


---


|  |  |  |
| --- | --- | --- |
| **Overview** \| **Package** \| **Class** \| **Use** \| **Tree** \| **Deprecated** \| **Index** \| **Help** | |  |
| **PREV CLASS**   **NEXT CLASS** | **FRAMES**    **NO FRAMES**     **All Classes** |  |
| SUMMARY: NESTED \| FIELD \| CONSTR \| METHOD | DETAIL: FIELD \| CONSTR \| METHOD |  |


---
